# Utilization of Never-Medicated Bipolar Disorder Patients towards Development and Validation of a Peripheral Biomarker Profile

**DOI:** 10.1371/journal.pone.0069082

**Published:** 2013-06-24

**Authors:** Catherine L. Clelland, Laura L. Read, Laura J. Panek, Robert H. Nadrich, Carter Bancroft, James D. Clelland

**Affiliations:** 1 Department of Pathology and Cell Biology, and Taub Institute for Research on Alzheimer’s Disease and the Aging Brain, Columbia University Medical Center, New York, New York, United States of America; 2 Department of Psychiatry, New York University, Langone Medical Center, New York, New York, United States of America; 3 Movement Disorders and Molecular Psychiatry. The Nathan Kline Institute for Psychiatric Research, Orangeburg, New York, United States of America; 4 Department of Psychiatry, Bellevue Hospital Center, New York, New York, United States of America; 5 Department of Structural and Chemical Biology, Mount Sinai School of Medicine, New York, New York, United States of America

## Abstract

There are currently no biological tests that differentiate patients with bipolar disorder (BPD) from healthy controls. While there is evidence that peripheral gene expression differences between patients and controls can be utilized as biomarkers for psychiatric illness, it is unclear whether current use or residual effects of antipsychotic and mood stabilizer medication drives much of the differential transcription. We therefore tested whether expression changes in first-episode, never-medicated BPD patients, can contribute to a biological classifier that is less influenced by medication and could potentially form a practicable biomarker assay for BPD. We employed microarray technology to measure global leukocyte gene expression in first-episode (n=3) and currently medicated BPD patients (n=26), and matched healthy controls (n=25). Following an initial feature selection of the microarray data, we developed a cross-validated 10-gene model that was able to correctly predict the diagnostic group of the training sample (26 medicated patients and 12 controls), with 89% sensitivity and 75% specificity (p<0.001). The 10-gene predictor was further explored via testing on an independent cohort consisting of three pairs of monozygotic twins discordant for BPD, plus the original enrichment sample cohort (the three never-medicated BPD patients and 13 matched control subjects), and a sample of experimental replicates (n=34). 83% of the independent test sample was correctly predicted, with a sensitivity of 67% and specificity of 100% (although this result did not reach statistical significance). Additionally, 88% of sample diagnostic classes were classified correctly for both the enrichment (p=0.015) and the replicate samples (p<0.001). We have developed a peripheral gene expression biomarker profile, that can classify healthy controls from patients with BPD receiving antipsychotic or mood stabilizing medication, which has both high sensitivity and specificity. Moreover, assay of three first-episode patients who had never received such medications, to first enrich the expression dataset for disease-related genes independent of medication effects, and then to test the 10-gene predictor, validates the peripheral biomarker approach for BPD.

## Introduction

BPD, characterized by periodic episodes of depression and mania, is a debilitating illness affecting approximately 2.6% of the US population [1]. BPD is usually diagnosed in the mid twenties [2], however prior to their first manic or hypomanic episode and subsequent diagnosis, patients often experience a long prodromal period characterized by symptoms of depression and anxiety [3,4]. Obtaining an accurate diagnosis during this early phase or first episode of mania is difficult; early adulthood or adolescent mania often manifests as atypical or dysphoric making it hard to identify, and patients frequently deny symptoms or self-report them inaccurately [5]. In addition, clinical assessment may altogether fail to take note of any past episodes of mania [6], and consequently levels of misdiagnosis are high [7–9]. For example, studies have documented levels of BPD underdiagnosis at approximately 40% [10,11], which persists even when a diagnosis is made after the first episode of mania [11], while more recently, BPD overdiagnosis was reported in 50% of patients retrospectively assessed and may arise in part due to the aggressive mood stabilizing drug marketing strategies [12]. These problems contribute to the often significant lag between an individual initially seeking treatment and then receiving a correct diagnosis [10,13,14], and subsequently have important treatment implications: A misdiagnosis of depression and the resultant use of antidepressants can induce mania and mood cycle alteration [15], while overdiagnosis can lead to unnecessary side effects from mood stabilizer treatment, failure to recommend the most effective treatment [13,16], and inappropriate care during, for example, the postpartum period [17].

The promise of an accurate test that could specifically differentiate, from their age-matched peers, patients with BPD early during the illness progression, has been clearly highlighted by studies reporting that early detection and initiation of mood stabilizing treatment in BPD patients is associated with more favorable clinical outcomes and reduced suicide risk [18–20]. Furthermore, both behavioral and neuroimaging studies have supported the utility of targeting diagnosis to the first episode of mania or before; patients both want and are more likely to respond to treatment at this stage [5] (and references therein), and the marked loss of brain volume associated with later illness recurrences is still relatively limited at the first-episode of mania [18] (and references therein).

There is evidence that peripheral gene expression differences in psychiatric disorder patients can be utilized as biomarkers of psychiatric illness. Initial studies of individuals with schizophrenia and bipolar disorder, using microarrays to measure global gene expression in peripheral blood leukocytes (PBLs) or peripheral mononuclear cells, have identified lists of genes significantly differentially expressed between patients and matched controls [21–23], that importantly could discriminate between diagnostic groups [21,23]. More recently, Niculescu and colleagues, also using microarray technology, have further explored the utility of peripheral gene expression as a biomarker for classification of symptoms, successfully identifying a panel of genes that could differentiate BPD patients based upon their mood state [24], or the severity of psychotic features in three cohorts of schizophrenia and schizoaffective disorder patients [25].

However, although this body of work suggests the promise of such a biomarker approach, patients utilized in these studies were ascertained later in their illness (mean age of BPD patients was 43 [21] and 45 years [23]), and all were receiving medication. Antipsychotic and mood stabilizer medication have a well known immunomodulatory effect [26–29], which likely impacts peripheral gene expression, and thus it is uncertain whether the biomarker profiles identified largely reflect the response to medications. Two recent and important papers have moved towards addressing this issue [30,31]. In a study of major depressive disorder, Spijker et al., identified a lipopolysaccharide stimulated lymphocyte signature that could discriminate 21 unmedicated major depressive disorder patients from 21 controls, that was confirmed with 71.4% specificity and 76.9% sensitivity in a replication sample of 13 patients and 14 controls [30]. Most recently, and using PBLs as a surrogate to identify pathways dysregulated in BPD, Beech et al., identified a list of 1180 genes significantly differentially expressed between 20 BPD patients (mean age of 38.4 years, of whom 9 were unmedicated), and 15 healthy controls [31], although this large gene dataset was not further refined into a biomarker signature and tested in follow-up cohorts.

Studies of first-episode BPD patients who have never received medication are more limited and also somewhat conflicting. For example, Padmos et al. reported striking data from three subjects who were, prior to onset, correctly predicted to develop a mood disorder based upon a monocyte gene expression signature developed upon medicated BPD patients [23]. However, in this study rates of mood disorder prediction of non-affected offspring of BPD patients and healthy children (45% and 19% respectively), were higher than expected [4,32], particularly considering the prevalence of mood disorders in control populations, and thus follow-up is required to assess mood symptom onset and validate this potentially promising biomarker profile. Conversely, a large study of first-episode BPD patients failed to confirm utility of potential blood biomarkers developed from assay of medicated patients [33].

We therefore set out to test whether leukocyte gene expression changes in first-episode, never-medicated BPD patients, when compared to healthy controls, can contribute to a biological classifier for bipolar disorder that is not induced by medication, and that has the potential to be developed into a practicable clinical biomarker assay without requiring that large transcript numbers be assayed per sample.

## Methods

### Subjects and Recruitment Procedure

#### Ethics Statement

The study was reviewed and approved by the institutional review boards of the Nathan Kline Institute, New York University Langone Medical Center, Rockland County Department of Mental Health and Columbia University Medical Center. Written informed consent was obtained from all subjects in accordance with the guidelines and regulations of the Nathan Kline Institute, New York University Langone Medical Center, and Rockland County Department of Mental Health institutional review boards, and also in accordance with New York State Office of Mental Health regulations and New York City Health and Hospitals Corporation regulations.

First-episode BPD (bipolar 1) patients were recruited from the Comprehensive Psychiatric Emergency Program at Bellevue Hospital Center, New York. To seek to reduce variability, all subjects recruited into the study were male. Recruitment was not restricted by ethnicity. To ensure the recruitment of first-episode patients prior to the initiation of medication, an on-call protocol was employed whereby a member of the study team was notified by the admitting psychiatrist immediately upon presentation of a potential subject at the Comprehensive Psychiatric Emergency Program. Medicated patients were recruited at Rockland Psychiatric Center, Orangeburg, NY, its associated outpatient facilities, and the Rockland County Pomona and Garnerville Clinics.

A Structured Clinical Interview for DSM IV Disorders (SCID) interview was conducted for all patients to confirm a diagnosis of BPD. Psychiatric and cognitive symptoms were also evaluated for first-episode patients, using the Brief Psychiatric Rating Scale, the Schedule for Assessment of Positive Symptoms, the Schedule for Assessment of Negative Symptoms, and the Mini-Mental State Examination.

Male control subjects were recruited from the local hospital communities and the NKI Volunteer Research Pool; a database of healthy control subjects also from the local community interested in participating in research studies. All controls completed a SCID non-patient interview.

General exclusion criteria for all subjects included current or recent-infectious diseases, recent physical trauma or surgery, and chronic immunosuppressant or anti-inflammatory medication use. For all subjects, a urine toxicology screen was performed, with urine obtained immediately prior to the collection of 15mls of whole blood from the anticubital vein.

Subjects recruited into this study were separated into two groups for analysis; a random 1:1 separation for control subjects, or for patients, based upon their medication status. For the first analysis step (feature selection for transcript enrichment, see below in *Data Analysis*) samples from the three never-medicated BPD patients and 13 controls were employed. Step two (the training, model-building and cross-validation paradigm), utilized samples from all medicated patients (n=26), and the remaining 12 control subjects (as described further below).

### Blood Sample Processing and Microarray Hybridization

Immediately after blood collection, PBLs were isolated by lysis of red cells, centrifugation and washing, according to standard protocols (Qiagen Inc., Valencia, CA, USA). Blood samples were immediately split into two; an “A” and replicate “B” sample (thus two separate RNA extractions, cDNA and cRNA syntheses and array hybridizations were performed). Purified white blood cell lysates were stored at -70^°^C prior to RNA extraction. The RNA quality of all samples was assessed using an 2100 Bioanalyzer (Agilent Technologies, Santa Clara, CA, USA), and an RNA Integrity Number (RIN)< 7.0 was used to determine sample degradation, and those samples discarded. 1ug of total RNA was employed as a template for cDNA synthesis, using an oligo-dT primer and Reverse Transcriptase enzyme (Life Technologies Inc., Carlsbad, CA, USA). Purified cDNA was employed as a template to generate biotin labeled cRNA, using an RNA Transcript labeling kit (Enzo Life Sciences Inc., Farmingdale, NY, USA). The quality of each final cRNA fragmented product was also assessed via the Bioanalyzer. All samples were considered of sufficient quality, and were thus fragmented and hybridized to U133plus2.0 microarrays (Affymetrix Inc., Santa Clara, CA, USA) at the Yale University Affymetrix facility (via the NIH Neuroscience Microarray Consortium project, http://arrayconsortium.tgen.org/), using standard protocols. MIAME compliant microarray data has been deposited into the NCBI GEO database, accession number GSE46449.

### Data Collection and Quality Control Assessment

To perform an initial assessment of sample quality, Affymetrix Cel files generated from the scanned microarrays were analyzed by the MAS5.0, algorithm in the Genechip Operating Software (GCOS-Affymetrix Inc, Santa Clara CA, USA), and expression intensity values obtained using a scaling factor of 100. The presence of contaminating globin transcripts and RNA degradation was evaluated by determining the percentage of present calls (the number of transcripts found to be expressed in leukocytes), and assessment of the 3’/5’ ratios of the control genes beta-actin and GAPDH. In our dataset, only three of the arrays had levels of low global expression as determined by percent present calls over the entire array of <40% (38.8%, 39.3% and 39.9% for the flagged samples), however all arrays were within a 10% range of present calls. No arrays were flagged for high 3’/5’ ratios of the control genes.

To evaluate the hybridization signal comparability within and across arrays, Cel files were also evaluated using ‘affy’, ‘simpleaffy’ and ‘affyPLM’ Bioconductor packages [34–37], through the R programming system [38]. Quality assessment measures were performed for randomized batches of microarrays (8-12 arrays per batch), including evaluation of the raw log-intensity signal distribution (via boxplots and density histograms), evaluation of intensity-dependent biases (via M-A plots), determination of spatial biases (via visualization of 2-D pseudo-images based on probe-level model residuals (the weights used by Robust Multichip Analysis (RMA) to address outliers in the data), and evaluation of probe-set homogeneity (via normalized unscaled standard error plots). No microarrays were flagged for issues with signal comparability.

For diagnostic group classification, Cel files were imported into the BRBArray Tools package version 3.8.1 [39], and converted to normalized intensity values using the Almost RMA (aRMA) algorithm [40], which uses a background correction on the Affymetrix perfect match data, then applies a quantile normalization and finally summarizes the probe set information by using Tukey’s median polish algorithm (with log transformation). aRMA (which is employed over RMA when large numbers of microarrays are present in the data collection process), has greater sensitivity and specificity for determining differential gene expression, when compared to, for example MAS5.0 [40].

The quality of sample replication was then assessed via three methods: unsupervised average-linked hierarchical clustering of the entire transcriptome, global pairwise analysis, and a calculation of the expression differences of a single control gene between replicates. Where a pair of replicates (the “A” sample and replicate “B” sample) failed assessment in at least two of the measures (replicate samples did not cluster together, and/or global pairwise comparison Spearman’s rho<0.985, and/or control gene expression log2A–log2B ± 2.3 SD from the mean), the replicate “B” sample was removed from further analysis.

### Data Collection of the Independent Test Cohort

We also investigated an independent test subject sample, collected by Matigian et al. [41], (data accessible at NCBI GEO database [42], accession GSE7036). The sample consisted of three medicated patients with BPD (bipolar 1 with psychotic features), and their three discordant twins. For each subject a lymphoblastoid cell line (LCL) had been established from whole blood [41], RNA extracted and processed for hybridization to Affymetrix U133plus2.0 microarrays (the same platform employed for our enrichment, training and replication samples). Expression data from a LCL was considered to be related to that of PBLs, thus representing a useful and completely independent sample on which to test the predictor gene-model. Cel files for the six subjects were downloaded from the GEO database, imported into BRB-Array Tools, and normalized expression values generated using aRMA as described above.

### Data Analysis

Analysis of subject group characteristics was conducted using SAS (Version 9.1). Group differences were tested using the Satterthwaite t-test or ANOVA with a correction for multiple testing (assuming normality of continuous variables), and using the χ^2^ or Fisher exact test where the expected cell size was <5 (categorical variables).

**Figure 1:**
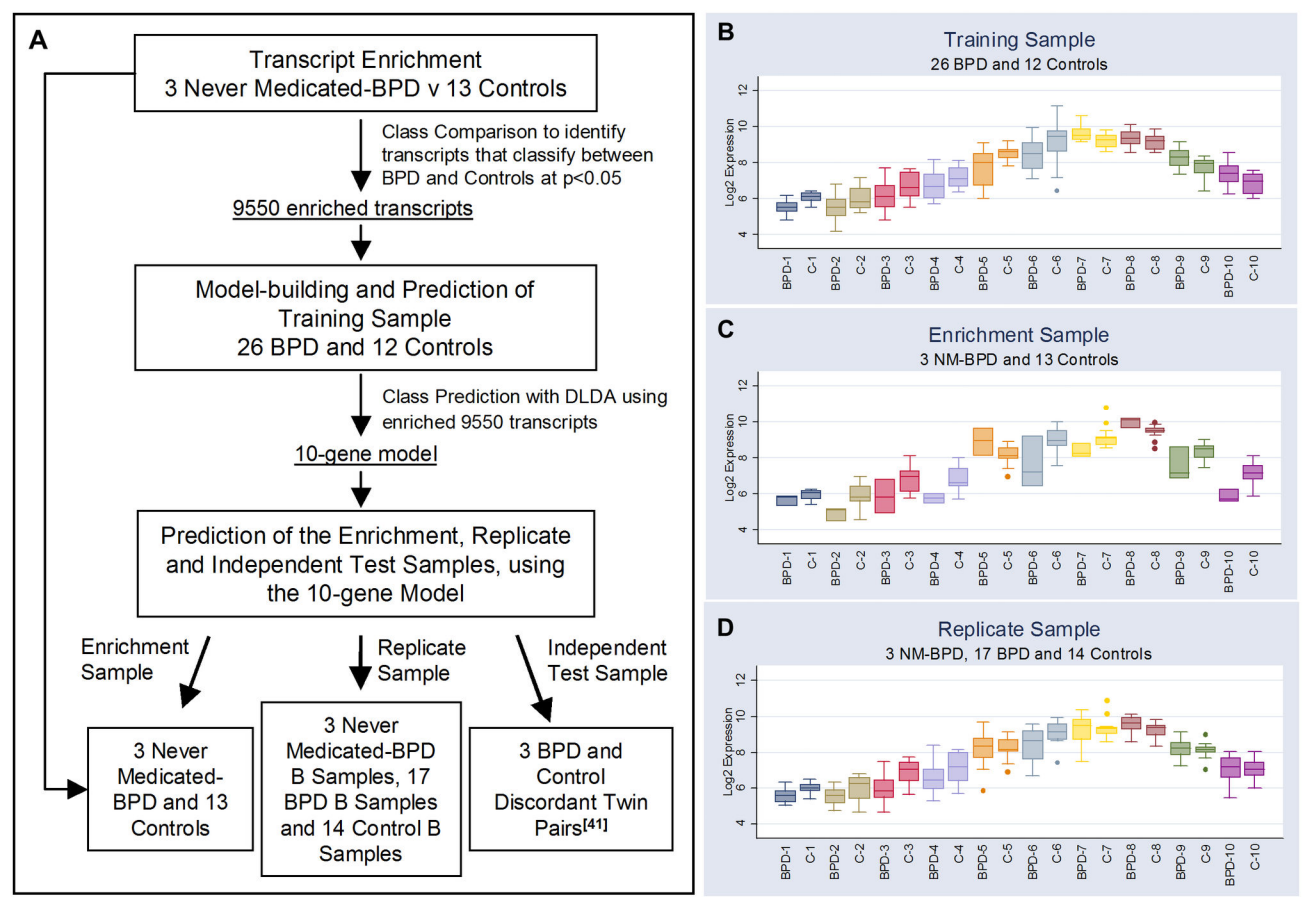
Experimental Strategy and Gene Expression Data for the 10-Gene Predictor Model. **A**: This panel depicts the study design and order of experimental analysis. **B**: Box plot of log expression data for each of the 10 genes in the model developed on the training sample cohort. The horizontal line within each box represents the group median (BPD or C) for each gene (genes 1-10). The box indicates the interquartile range (IQR), the whiskers represent 25th and 75th percentiles of the data, and outliers are depicted as circles. 84% of the classes were correctly predicted using this 10-gene model in a DLDA (p<0.001), with 89% sensitivity and 75% specificity. **C**: Log expression data of the enrichment sample cohort for each of the 10 genes. 88% of the sample were correctly predicted, with a sensitivity of 67% and specificity of 92% (p=0.015). **D**: Log expression data of the replicate samples for each of the 10 genes. Six of the control subjects with B samples in the replication sample were originally randomly sampled to the training sample, and eight originally randomly sampled to the enrichment sample. 88% of the replicate samples were correctly predicted, with a sensitivity of 90% and specificity of 86% (p<0.001). Concordance between replicates (for each of the 34 individual subjects with a replicate sample, class prediction of the A replicate = class prediction of the B replicate), was greater than 85%. **Key**: BPD= bipolar disorder patients; C=control subjects; NM= Never-Medicated, DLDA= Diagonal Linear Discriminant Analysis. **Key to Gene List (Probeset ID, Gene Symbol, description)**: Gene 1: 212282_at, TMEM97, transmembrane protein 97. Gene 2: 236769_at, LOCI158402, Hypothetical protein LOCI158402. Gene 3: 231798_at, NOG, noggin. Gene 4: 1568983_a_at, unknown transcript, unknown. Gene 5: 1560527_at, NF-E4, transcription factor NF-E4. Gene 6: 208304_at, CCR3, chemokine (CC motif) receptor 3. Gene 7: 230000_at, RNF213, ring finger protein 213. Gene 8: 225252_at, SRXN1, sulfiredoxin 1 homolog. Gene 9: 210425_x_at, GOLGA8B, golgi autoantigen, golgin subfamily a, 8B. Gene 10: 227884_at, TAF15, TAF15 RNA polymerase II, TATA box binding protein (TBP)- associated factor, 68kDa.

Figure 1A depicts the data analysis strategy. A class comparison algorithm was initially employed in a non-conservative feature selection; to both remove irrelevant and redundant features from the data, and importantly to enrich for disease-related differential transcription not driven by medication effects. The algorithm was set to identify transcripts that were differentially expressed among the two classes of the enrichment sample cohort (the three never medicated BPD subjects plus 13 of the control subjects selected at random), using a random-variance t-test [43]. Transcripts were included if their unadjusted p-value was less than 0.05.

Using the enriched transcript set (n=9550), we developed an expression model to predict the class of subjects in the training sample cohort (26 medicated patients and the remaining 12 controls). The BRB-Array Tools package simultaneously performs and reports model statistics based upon seven well-known classification algorithms (for BRB-Array Tools Users Guide see 39). Each classifier was set to incorporate genes based upon a Support Vector Machine Recursive Feature Elimination (SVM RFE), which involves an iterative process of removing transcripts with the lowest weight for each prediction method, until the desired number of transcripts are left (using the default setting of n=10). We reasoned that use of an RFE would ultimately generate a model that, if sensitive and specific, could be developed into a realistic clinical biomarker test assaying only a small number of gene transcripts. Class prediction of the enrichment, replicate and test cohorts was carried out as described above, except that the classifiers incorporated only the 10 genes from the original prediction model.

The 10-gene model was validated for the training cohort using a leave-one-out cross-validation (LOOCV) design, as described by Simon et al. [44],). For studies with small n, it has been suggested that validation of prediction error rates using a simple split-sample design has the potential for bias [44–46]. LOOCV is an alternative and robust form of error rate validation that works well for small samples [45], and encompasses an iterative process, whereby one sample at a time is omitted from the analysis. For the sample omitted, the entire analysis is repeated from scratch, including the RFE determination of genes predicted from the n-1 training cohort. The 10-gene predictor is then applied to the sample that was omitted, and the LOOCV reports whether that prediction was correct or not. The process of omission, model building, and testing, is repeated for every sample [39].

For all cohorts (training, enrichment, replicate, and test) we also evaluated whether the error rate estimate for our model was significantly less than one would expect by chance, based upon 1000 random permutations. The significance level is the proportion of the random permutations that gave an error rate no greater than the error rate obtained with the real data. Thus, for example, when p=0.015, only 1.5% of the 1000 permutations, by chance, gave a class prediction equivalent to or better than the one obtained on the actual dataset.

### Concordances with Brain Expression

CNS expression levels of the 10 genes in the predictor were obtained from the brain collection microarray studies published by the Stanley Medical Research Institute (SMRI) [47]. The brain collections consist of the Consortium Collection (BA46/10, BA6, BA8/9 and cerebellum from 15 subjects with BPD and 15 unaffected controls), and the Array Collection (BA46 tissue from 35 BPD and 35 controls). The published data summarizes global gene expression from both collections, over 12 individual microarray studies and across 6 different microarray platforms. We considered that due to the controlled nature of the brain sampling and study design [47], use of this gene expression data would be robust with limited experimental error. Gene expression was considered concordant between brain and blood if the direction of expression between BPD and controls was consistent between the SMRI data and that of the replication sample cohort (as this sample contained all subject groups), with SMRI p-values reported.

## Results

Three first-episode, never-medicated BPD patients and 26 medicated BPD patients were recruited into the study. 25 healthy control subjects were also recruited (see Table 1). As shown in Table 1 there were no differences between subject groups on collected demographic variables, except age: there was a trend for medicated patients to be older at the time of blood draw when compared to control subjects. Medicated patients received a wide-range of antipsychotic, mood stabilizing, and antidepressant medications (Table 2). A SCID non-patient interview was conducted for all controls, and none reported symptoms from modules A-D, except for two control subjects, one of whom had a single episode of depressive disorder not otherwise specified, and the other a brief past depressive episode. A urine tox screen was performed for all subjects: only one subject, a first-episode patient, was found to be positive (for Tetrahydrocannabinol). This first-episode patient also reported taking 60mg of Adderall (a psycho-stimulant), daily, two weeks prior to admission and participation in the study. The characteristics of the three discordant twin pairs, employed in the independent test set and taken from [41], are shown in Table 3.

**Table 1:** Characteristics of Study Subjects.

**Characteristic**	**NM-BPD**	**BPD**	**Controls**
**n**	3	26	25
**Ethnicity, % (n)**			
African American	0 (0)	16 (4)	28 (7)
Caucasian	67 (2)	80 (21)	60 (15)
Hispanic	0 (0)	4 (1)	4 (1)
Asian	33 (1)	0 (0)	8 (2)
**Age, years, mean (SD)**	33.3 (4.0)	40.8* (14.4)	32.5 (9.7)
**Age at First Hospitalization, years**			
Mean (SD)	33.3 (4.0)	25.0 (13.13)	
**Treatment Setting, % (n)^a^**			
Inpatient	100 (3)	53.9 (14)	
Outpatient	0 (0)	46.1 (12)	
**With Psychotic Features, %** (n)	100 (3)	42.4 (11)	0 (0)
**BPRS^b^ Total Symptoms**, mean (SD)	39 (5.3)		
**SAPS^c^ Total Symptoms**, mean (SD)	29.7 (16.3)		
**SANS^d^ Total Symptoms**, mean (SD)	15.7 (5.5)		
**MMSE^e^ Total Symptoms**, mean (SD)	28.7 (0.6)		

^*^ F_(2,51)_=3.11, p 0.053. There were no other significant differences of demographic variables across the three groups (ethnicity), or patient groups (age at first hospitalization and treatment setting).

a Outpatient includes outpatients living on hospital grounds and in the community

b Brief Psychiatric Rating Scale

c Schedule for Assessment of Positive Symptoms

d Schedule for Assessment of Negative Symptoms

e Mini-Mental State Examination

**Table 2:** Medication Profiles of Study Subjects.

**Medication**	**NM-BPD**	**BPD**	**Controls**
**n**	3	26	25
**Antipsychotics, % (n)**			
None	100 (3)	27 (7)	100 (25)
Atypical Only		50 (13)	
Typical Only		15 (4)	
Both		8 (2)	
**Dose, Median (IQR)**			
CPZ equivalents^a^	0 (0)	479.1 (399.8)	0 (0)
Aripiprazole (n=3)		3, 20 (20)	
Clozapine (n=1)		300 (0)	
Haloperidol (n=1)		20 (0)	
Olanzapine (n=4)		25 (15)	
Quetiapine (n=9)		450 (100)	
Risperidone (n=3)		5.4 (2.9)	
Ziprasidone (n=2)		160 (0)	
**Mood Stabilizers**, % (n)	0 (0)	81 (21)	0 (0)
**Dose, Median (IQR)**			
Lithium (n=9)		750 (300)	
Divalproex (n=6)		1125 (1000)	
Gabapentin (n=3)		900 (600)	
Lamotrigine (n=6)		175 (50)	
Oxcarbazepine (n=1)		1200 (0)	
Valproic Acid (n=1)		2000 (0)	
**Antidepressants**, % (n)	0 (0)	46.2 (12)	0 (0)
**Dose, median (IQR)**			
Bupropion (n=1)		200 (0)	
Citralopram (n=1)		20 (0)	
Venlafaxine (n=2)		112.5 (75)	
Fluoxetine (n=3)		20 (20)	
Sertraline HCl (n=3)		100 (150)	
Trazodone (n=2)		125 (50)	

a Chlorpromazine-equivalent dose

**Table 3:** Characteristics of the Monozygotic twin pairs [41].

**Characteristic**	**Twin pair 1**	**Twin Pair 2**	**Twin Pair 3**
**n**	2	2	2
**Age at onset^a^**	19	19	18
**Age^b^**	55	23	38
**Gender**	female	male	female
**Medication^a^**	Lithium and Citralopram	Lithium and Citralopram	Sodium valproate

a BPD twin only

b Age at blood draw

A sufficient blood sample was obtained from 41 subjects (including all first-episode patients) for complete sample replication. Thus, ninety-five leukocyte samples (the 41 subjects with replicate samples, plus an additional thirteen subject samples) were processed to completion and hybridized to U133plus2 microarrays. All samples passed checks for RNA quality and quality controls within and between arrays. The quality of sample replication was also assessed: Where a pair of replicates (A and B replicated samples from the 41 subjects) failed assessment in at least two of the measures, the B sample was removed from further analysis (n=0 first-episode patients, n=3 medicated patients, and n=4 controls).

In the field of computationally extensive machine learning, the need to reduce irrelevant complexity of the data is well known. Class prediction [48], a technique based upon use of supervised leaning algorithms that can utilize microarray data to classify between two diagnostic classes, is no exception. Thus, to reduce dimensionality of the dataset we performed a simple feature selection: A class comparison analysis [48] was employed to refine a subgroup of transcripts significantly differentially expressed (p<0.05) between the classes of our “enrichment” subject group, which consisted of the never-medicated BPD patients and a subset of the control subjects (see Figure 1A). We hypothesized that the resultant transcript list (n=9550) would be enriched for disease-related differentially expressed transcripts that were not driven by medication effects. The enriched gene set was then employed in a class prediction of subjects in the separate training sample: 26 medicated BPD patients and the remaining 12 control subjects. Specifically, we developed a gene prediction model that utilized a SVM-RFE algorithm to identify 10 genes that could predict the class of the training samples. Expression levels of the 10-gene predictor are shown in Figure 1B. For validation of our model, and in order to avoid reducing the size of our training sample further (as would be required for a split-sample validation), we employed a LOOCV technique. The cross-validated error rate, ranged from 0.18 to 0.13 based upon the classification algorithm employed, and all were unlikely to occur by chance: Compound Covariate Predictor (0.18, p=0.004); Diagonal Linear Discriminant Analysis (0.16, p<0.001); 1-Nearest Neighbour (0.13, p,0.001); 3-Nearest Neighbours (0.13, p<0.001); Nearest Centroid Correct (0.16, p=0.001); Support Vector Machine (0.18, p=0.004); and Bayesian Compound Covariate Predictor (0.17, p=0.004).

Diagonal Linear Discriminant Analysis (DLDA), applies a discriminate transformation to the expression data, using a weighted linear combination of log-intensities for genes, whereby genes in which larger values of the log-ratio pre-dispose to class 2 and have weights of one sign, whereas genes in which larger values of the log-ratios pre-dispose to class 1 have weights of the opposite sign. The univariate t-statistics for comparing the classes are used as the weights (thus by definition, more weight is given to genes with a high-signal-to-noise-ratio), and employed in the RFE. It has been suggested that because DLDA ignores correlations among genes it is particularly useful in avoiding data overfitting, with the lowest reported cross-validated error rates following direct comparison of multiple prediction algorithms [49]. Based upon this attribute, we chose to focus further exploration of the 10-gene predictor using only the DLDA classifier. DLDA correctly predicted the diagnostic group of 84% of the training sample set (see Table 4). Interestingly, the two control subjects who reported depressive episodes were both predicted to belong to the bipolar group, and after removing these subjects, the predictor yielded a sensitivity and specificity of 89% and 90%, respectively. We also examined the effect of utilizing the SVM-RFE algorithm to identify a predictive model containing n=5, 15, 20, 25 to n=40 genes. None reached the level of sensitivity and specificity achieved by the initial and default n=10 model, and so only this model was carried forward in the analysis.

**Table 4:** Performance of the 10-Gene Predictor in a DLDA.

**Sample**	**BPD Correctly Predicted / n**	**Control Correctly Predicted / n**	**Total Correctly Predicted**	**Sensitivity**	**Specificity**	**LOOCV^a^**
**Training (n=38)**	23/26	9/12	84%	89%	75%	p<0.001
**Enrichment (n=16)**	2/3	12/13	88%	67%	92%	p=0.015
**Replication (n=34)**	18/20	12/14	88%	90%	86%	p<0.001
**Test (n=6)**	2/3	3/3	83%	67%	100%	p=0.18

a Leave-One-Out-Cross-Validation permutation p-value, based upon 1000 permutations.

We tested our predictor by employing the 10-gene model in a DLDA of the original enrichment sample dataset (n=3 first-episode patients and n=13 controls), and in addition, to assess experimental reproducibility, the replicate samples (the B replicates from 34 subject): 88% of sample diagnostic classes were correctly predicted for both the enrichment and replicate datasets (p=0.015 and p<0.001 respectively, see Figure 1C and 1D), and the concordance between predictions of the replicate samples was greater than 85%.

Finally, we performed exploratory validation of our 10-gene predictor using data from an independent test sample: LCL expression data obtained from three pairs of monozygotic twins discordant for BPD [41]. 83% of these samples were correctly predicted with a specificity of 100% and sensitivity of 67% (only one of the 6 subjects, the BPD twin from pair 3, was incorrectly predicted). However, 18% of the 1000 random permutations correctly predicted five or more of the samples (LOOCV permutation p=0.18). This elevated prediction error-rate may highlight differences between the tissue sources (such as the need to transform the cells) that could introduce gene expression variation in the predictor genes and mask the BPD biomarker profile, or may reflect the small sample size.

The 10 genes from the predictor are summarized in Table 5. Interestingly, 70% map to loci previously linked to BPD [50]. To examine the implications of this finding, we randomly chose and mapped to the human genome 500 transcripts from the microarray. Although 203 (40%) also mapped to linkage regions, there was a trend for genes in the predictor to be associated with BPD loci, compared to the 500 randomly permuted genes (p=0.061). As also shown in Table 5, of the genes that were expressed in the brain and measured in the SMRI microarray studies (n=6), four were co-regulated between brain and blood, which is consistent with previous data reporting a moderate correlation between global CNS and blood gene expression, with approximately 50% of psychiatric illness relevant genes tested expressed in both tissues [51].

**Table 5:** The 10-gene Predictor.

**Gene Number^a^**	**Affymetrix probeset ID**	**Gene Symbol**	**Description**	**Chromosome location^b^**	**Concordant Brain expression^c^**
Gene 1:	212282_at	TMEM97	transmembrane protein 97	17q11.2*	Downregulated in BPD: Consortium (p=0.025)^d^
Gene 2:	236769_at	LOCI158402	Hypothetical protein LOCI158402	9q32*	Downregulated in BPD: Consortium (p=0.23)
Gene 3:	231798_at	NOG	noggin	17q22	No change^e^
Gene 4:	1568983_a_at	unknown	Unknown transcript.	15q21.2	Data not available
Gene 5:	1560527_at	NF-E4	transcription factor NF-E4	7q22.1*	Data not available
Gene 6:	208304_at	CCR3	chemokine (CC motif) receptor 3	3p21.3*	Downregulated in BPD: Array (p=0.15)^e^
Gene 7:	230000_at	RNF213	ring finger protein 213	17q25.3*	No change
Gene 8:	225252_at	SRXN1	sulfiredoxin 1 homolog	20p13*	Not expressed
Gene 9:	210425_x_at	GOLGA8B	golgi autoantigen	15q14	Data not available
Gene 10:	227884_at	TAF15	TAF15 RNA polymerase II	17q11.1-q11.2*	Upregulated in BPD: Array (p=0.0108)

a From Figure 1b–1d

b * denotes loci previously linked to BPD [48].

c Data from the Replication sample compared to microarray expression data from the SMRI [46]. Only the brain collection(s) with concordant expression are noted, no change between BPD patients and controls was observed for the collections not described, except for Gene 1

d : the collections showed an opposite trend of differential expression, array collection upregulated in BPD, p 0.072).

e SMRI data suggests very low expression in the collections examined.

## Discussion

Recognizing the need for a biological assay that could help diagnose BPD and potentially define susceptibility at an early stage, we and others [21,23], have successfully developed a peripheral gene expression biomarker profile that can accurately classify healthy controls from patients with BPD who are currently receiving or have previously received antipsychotic or mood stabilizing medication, which has both high sensitivity and specificity, with a low cross-validated prediction error rate.

Important features of a biomarker include high sensitivity and specificity, with the need to be able to test and validate the predictor on both replication and independent cohorts. Moreover, the expression biomarker must not be dependent on other group differences, such as medication status; to date the majority of gene expression class prediction studies for psychiatric illness have identified gene profiles that classify between medicated patients and unmedicated control subjects. Therefore, utilizing the 10-gene predictor trained upon medicated patients and controls, we explored whether we were also able to predict the class of the enrichment cohort, which included the first-episode patients and a separate group of controls. We observed high class prediction specificity (92.3%), with a sensitivity of 66.6%, and this result was unlikely to occur by chance (p=0.015). One caveat to this approach, and due to our small sample, is the use of the enrichment set to both perform the feature section (reducing the dataset from 54,675 to 9550 transcripts), and then to test the 10-transcript model developed upon the training sample. While the purpose of the feature selection was to enrich for disease-related differential expression that was not driven by medication status, the downstream testing of the 10-gene model in this cohort should not be considered completely independent from the model-training process. Thus, even though the 10-gene model represents only 0.1% of the total enriched dataset, the error rates may be positively biased due to the use of incomplete cross-validation [46]. Nonetheless, assay of first-episode patients who have never received antipsychotic or mood stabilizing medication (the first transcriptome level study of never-medicated BPD patients) supports the peripheral biomarker approach for early BPD diagnosis.

To assess the ability to replicate our findings we independently assayed each subject sample in duplicate. We correctly identified the diagnostic class of 88% of the subjects in the replicate cohort with a very low predicted error. Only five samples did not give identical class predictions across replicates. While this result was not unexpected, the high level of experimental replication suggests that the PBL gene predictor can resist technical variation.

Validation of gene predictor models is an important step during determination of the non-biased error rate. This can be achieved using a cross-validation design (such as LOOCV), or alternatively it is also common in the field of class prediction to employ a split-sample design, whereby one group of subject samples are employed in training of the predictor, and a completely independent group employed to singularly test the model. While this may be a powerful approach, a meaningful, unbiased predictor requires that both training and test sets are sufficient in size as to be representative of their original population distributions; for example, Molinaro et al., showed that only from analysis of 120 samples or more did the rates of error bias from the split-sample design reduce to rates observed using LOOCV [45], and the spilt-sample approach is not recommended for cohorts of size less than 150 [52]. Thus, due to our sample size, further separation of patients and control samples to derive a test sample would likely add bias to the predicted error rates [45]. Therefore, we obtained an independent microarray dataset consisting of transformed lymphocyte expression data from three pairs of monozygotic twins discordant for BPD [41]. Methods of subject ascertainment, recruitment, blood sampling and processing varied dramatically between our study and that reported by Matigian et al., however only 1 subject from that study (a BPD twin) was incorrectly assigned to their diagnostic group using our 10-gene predictor (83% correct assignment, 100% specificity, and 66.6% sensitivity). Although this is a promising result, the non-significant LOOCV does not allow us to rule out that chance contributed to this result, and care must be taken when interpreting error rates from small validation sets [44]. Additional analyses of larger independent test sets of leukocyte expression, particularly expression data obtained from first-episode never-medicated patients, is warranted to confirm our findings and we consider the 10-gene classifier as only a first iteration of a predictive model for BPD diagnosis.

Even with the knowledge that a final biomarker profile for BPD needs further development, the ten genes in this reported model are potentially relevant to BPD biomarker diagnosis; the majority are linked with BPD and when expressed, are co-regulated in the CNS. Work by others has further highlighted their potential importance to neuropsychiatric disorders, including BPD. For example, Noggin (NOG, Gene 3 of the predictor) is an inhibitor of bone morphogenetic proteins, is expressed in the CNS [53], and is involved in multiple functions both during development and in the adult such that knockout animals have defects in neural tube fusion and joint formation [54], and noggin-mediated inhibition of BMP signaling has been reported to induce improvements in neurogenesis and cognition in mice [55]. The chemokine (CC motif) receptor 3 transcript (Gene 6), which encodes a G-protein-coupled receptor [56] was previously found to be downregulated in postmortem dorsolateral prefrontal cortex tissue from patients with bipolar disorder [57], and peripheral blood expression of RNF213 (Gene 7, the ring finger protein 213) was found to correlate with response to the atypical neuroleptic risperidone by children with autism spectrum disorders [58]. Supporting our findings, Beech et al., who identified genes differentially expressed between BPD patients and controls, also found upregulation of the SRXN1 gene (Gene 8, sulfiredoxin 1 homolog, whose encoded product reduces oxidant stress-generated cysteine-sulfinic acid), in PBLs of BPD patients, including unmedicated patients, when compared to controls [31].

Regarding limitations, there were a number of caveats to this study, such as the potential for confounding effects of age on the biomarker profile, and the use of only males in the study reducing generalizability. With regards to age, although there was a trend for medicated patients to be older than controls, with age thus potentially driving the medicated patient trained predictor, first-episode patients were not different in age to controls, and the ages of the independent cohort were matched due to the nature of the twin study. Concerning generalizability, four of the independent sample twin cohort were female, of which one, a BPD patient, was the only subject in this cohort to be incorrectly classified. Further study of PBL expression in females is warranted however, to determine if gender differences contributed to this result or if the incorrect prediction simply arose due to the different tissue sources and experimental procedures, such as the transformation process to obtain LCLs from this subject.

In general, the 10-gene predictor had high rates of specificity across study cohorts, suggesting that a positive prediction of BPD translates to a high probability of the presence of the illness. In clinical practice such a test would, for example, reduce levels of side effects from unnecessary mood stabilizer treatment due to overdiagnosis, but may result in missed opportunities to treat patients due to the lower sensitivity of the assay. Continuing this work, via the necessary assay of additional first-episode BPD patients and matched controls for model validation on completely independent test cohorts, and also cohorts of patients with unipolar depression, may help to develop and define the true utility of this PBL gene predictor.
